# MRI derived hippocampal asymmetry identifies hippocampal sclerosis in epilepsy surgical specimens

**DOI:** 10.1093/braincomms/fcag320

**Published:** 2026-08-21

**Authors:** Philbert Ndagijimana, Daniel Brennan, Russell T Shinohara, James J Gugger

**Affiliations:** Department of Neurology, University of Rochester, School of Medicine and Dentistry, Rochester, NY, USA; Department of Neurology, University of Pennsylvania, Perelman School of Medicine, Philadelphia, PA, USA; Department of Biostatistics, Epidemiology, and Informatics, Penn Statistics in Imaging and Visualization Center, Center for AI and Data Science for Integrated Diagnostics, University of Pennsylvania, Perelman School of Medicine, Philadelphia, PA, USA; Department of Neurology, University of Rochester, School of Medicine and Dentistry, Rochester, NY, USA; Department of Neurology, University of Pennsylvania, Perelman School of Medicine, Philadelphia, PA, USA

**Keywords:** asymmetry index, hippocampal sclerosis, epilepsy, normative modelling, morphometry

## Abstract

Hippocampal sclerosis is the most common histopathological diagnosis in epilepsy surgical specimens. Although hippocampal sclerosis can be identified *in vivo* with brain MRI, it cannot be detected by expert qualitative review in as many as 50% of cases. Quantitative features derived from structural brain MRI, such as hippocampal shape, volume, and asymmetry features, show promise in identification of hippocampal sclerosis *in vivo* but have had limited translation to clinical practice partly due to the need for large normative cohorts for estimation of hippocampal pathology. Using the Imaging Database for Epilepsy And Surgery cohort, which includes brain MRI scans from 442 individuals with drug-resistant focal epilepsy who underwent epilepsy surgery and 100 healthy controls acquired on multiple scanners, we evaluated the diagnostic performance of the hippocampal asymmetry index calculated using only the participant’s hippocampal volumes against a gold standard histopathological diagnosis of hippocampal sclerosis. The hippocampal asymmetry index had high diagnostic performance (area under the curve for left hippocampal sclerosis = 0.95 (95% confidence interval 0.92–0.97); right hippocampal sclerosis = 0.96 (95% confidence interval 0.94–0.98) and was similar regardless of segmentation algorithm, data harmonization, and use of normative data. To facilitate validation, we provide an open-source software tool for the calculation of the hippocampal asymmetry index.

## Teaser

Using clinically acquired brain MRI from 442 individuals with focal epilepsy, Ndagijimana *et al*. demonstrate that hippocampal volumetric asymmetry is a robust marker of pathologically confirmed hippocampal sclerosis. The asymmetry index is calculated using individual patient data only and does not require normative data. They release opensource software to facilitate validation.

## Graphical Abstract

**Graphical Abstract:**
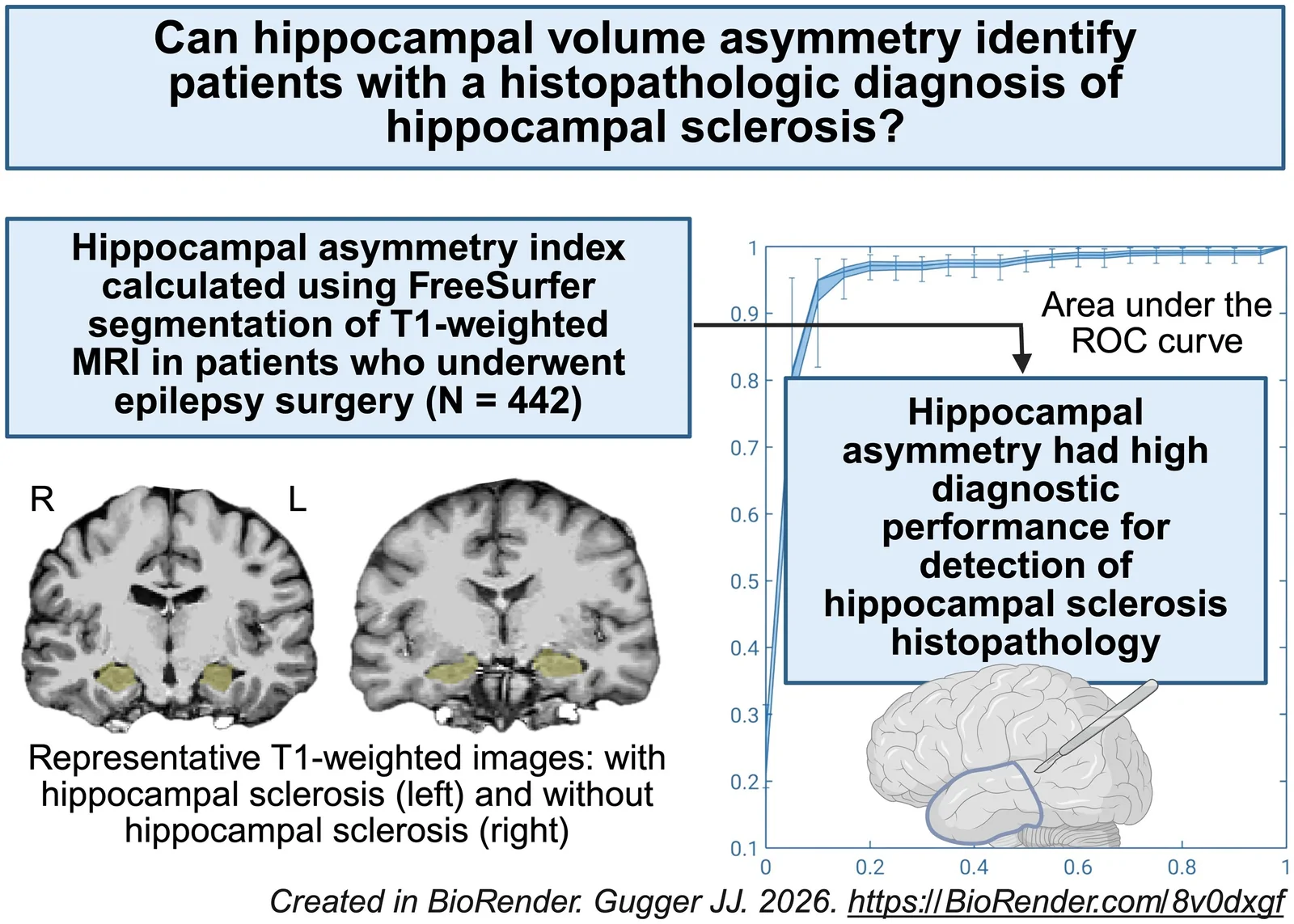

## Introduction

In contemporary studies of focal epilepsy, a causative lesion is identified in only 12–16% of patients.^1,2^ While this may be inconsequential for those who become seizure-free with antiseizure medication, about a third of patients continue to have seizures despite appropriately selected antiseizure medication.^3^ In these patients, epilepsy surgery can lead to seizure freedom, but the odds of successful surgery hinge on whether an epileptogenic lesion can be identified on pre-operative brain MRI.^4^ Furthermore, patients without an epileptogenic lesion identified on MRI generally require more diagnostic testing (including seizure localization with invasive intracranial EEG) before they are considered candidates for surgery and frequently do not undergo surgery because of perceived low chances of seizure freedom.^4^ Taken together, there is a critical need to increase the diagnostic yield of brain imaging modalities in detecting epileptogenic lesions in focal epilepsy.

Despite the commonality of nonlesional MRI in focal epilepsy, hippocampal sclerosis (HS) remains the most common histopathological diagnosis in epilepsy surgical specimens.^5^ Although brain MRI is the only way to diagnose HS *in vivo*, qualitative review fails to identify histopathologically confirmed HS in 30–50% of cases.^6^ Recent efforts at automatic detection of HS using clinically acquired brain MRI report high accuracy in detection of radiologically^7^ and pathologically^8^ identified HS by leveraging large multisite datasets consisting of epilepsy patients and controls. Such studies utilize morphometric data such as hippocampal shape, volume, and the asymmetry of these features, which are then compared to a large normative cohort to generate patient-specific z-scores in an approach known as normative modelling. Unfortunately, clinical translation of these tools has been limited, in part due to the requirement of large normative (healthy control) cohorts to estimate hippocampal pathology. Complicating matters, large cohorts are often acquired at multiple study sites where differences in scanner manufacturer, imaging protocol and scanning parameters introduce unwanted site (or batch) effects.^9^ The asymmetry index or laterality index is a quantitative tool that leverages the contralateral homologue as the control, and is widely used in the field of language lateralization with functional MRI. However, it remains unknown whether HS can be detected with accuracy suitable for clinical translation using asymmetry-based features without comparison to normative multisite data.

The purpose of this study is to determine if a participant-specific quantitative feature derived exclusively from clinically acquired 3D T1-weighted brain MRI can be used to detect HS with high confidence without the need for multisite normative data. We evaluated the utility of a measure of hippocampal volumetric asymmetry derived from clinically acquired brain MRI data in the identification of a pathologic diagnosis of HS among patients who underwent epilepsy surgery for drug-resistant seizures. We first show the presence of unwanted site effects in hippocampal volumes, but not in the asymmetry index. We found that the hippocampal asymmetry index had high diagnostic performance for detection of a histopathologic diagnosis of HS, a finding that was robust to different segmentation algorithms, use of data harmonization, and use of normative data. To facilitate validation, we provide an open-source software tool for calculation of hippocampal asymmetry index.

## Materials and methods

### Cohort description

This study utilized the open-source Imaging Database for Epilepsy And Surgery (IDEAS) dataset, which includes preprocessed MRI scans from 442 individuals with drug-resistant focal epilepsy who underwent epilepsy surgery as well as 100 healthy controls.^10^ This includes 215 (49%) patients with a pathologic diagnosis of HS, with 122 (28%) undergoing left-sided and 93 (21%) right-sided surgeries, respectively. All available demographic information is summarized in Supplementary Table 1. Pre-operative 3D T1-weighted images were acquired on three different GE scanners. More information on scanner model and voxel volume is provided in Supplementary Table 2.

### Image processing pipeline

As described in the original publication,^10^ pre-operative T1-weighted scans were processed with FreeSurfer (v7.3.2) using the recon-all processing stream followed by hippocampal subfield segmentation.^11^ Outputs were visually inspected following established quality control procedures as described in the original publication. Mean volumes of the left and right hippocampus as well as estimated intracranial volume were derived from the automatic subcortical segmentation (aseg). For sensitivity analyses, volumes of the left and right whole hippocampus were derived from the hippocampal subfield segmentation module. Three sets of features were separately calculated from hippocampal segmentations: (1) hippocampal asymmetry index, (2) normative hippocampal asymmetry index, and (3) normative hippocampal volumes.

### Hippocampal asymmetry Index

To assess for left-right asymmetry in hippocampal volumes, we calculated the asymmetry index (AI) using the following formula:


(1) $$\mathrm{AI}\;=\;\frac{L\;-\;R}{L\;+\;R}$$

Where L corresponds to the volume of the left hippocampus, and R corresponds to the volume of the right hippocampus.

### Normative hippocampal asymmetry index

To account for normal hemispheric asymmetry in hippocampal volumes and the potential influences of age and sex,^12^ we constructed generalized additive models (GAM) using observations from controls:


(2) AI∼1+age+sex

We then calculated a z-score for each patient using the residuals (the difference between observed and predicted values) and the mean and standard deviation of the residuals of the control cohort. These patient-specific z-scores represent the normative AI controlling for the effects of age and sex.

### Normative hippocampal volume

As in our prior work,^13^ we also calculated normative hippocampal volumes. We fit a GAM in controls separately for both left and right hippocampal volumes:


(3) volume∼1+age+sex+ICV

Using the residual approach described above, age, sex, and intracranial volume were then regressed out, resulting in patient-specific z-scores for both left and right hippocampus.

### Statistical analysis

#### Harmonization

To assess for confounds introduced by the use of different MRI scanners and imaging protocols, volume- and asymmetry-based features were harmonized using ComBat, a validated batch effect correction tool.^14^ Biological effects of interest included in the model were age, sex, and group (i.e. healthy control, epilepsy with right HS, epilepsy with left HS, and epilepsy without HS). The batch variable included categories representing each unique combination of scanner model and 3D T1-weighted image voxel volume. Batches with fewer than four participants (*n* = 9) were excluded. We assessed for the presence of batch effects in volumes and asymmetry indices by fitting ANOVA models on both unharmonized and harmonized features:


(4) feature∼1+age+sex+disease+batch

Where feature represents asymmetry index, left hippocampus volume, or right hippocampus volume, and disease represents the four-level group variable used in harmonization. A *P*-value < 0.05 for the batch factor is interpreted as evidence of batch effect. To assess differences in variance of features across batches, we performed a Bartlett’s test on the residuals from each of the above ANOVA models.

#### Diagnostic performance

We evaluated the diagnostic performance of each of the above-described unharmonized features (i.e. asymmetry index, normative asymmetry index, and ipsilateral normative volumes) in identifying cases with a pathologic diagnosis of HS using receiver operating characteristic (ROC) curves. Histopathologic labels for patients were categorized as either left HS (*N* = 122), right HS (*N* = 93), or other pathology (*N* = 227). Controls were not included. We constructed separate ROC curves for the detection of right and left HS, where the other two histopathologic labels were considered the negative class. Optimal operating points were computed using the *perfcurve* function in MATLAB, which selects the ROC point based on a cost-sensitive tangent-line criterion. Area under the curve, sensitivity, and specificity were calculated with pointwise 95% confidence intervals with 1000 bootstrap replicates. Five-fold cross-validation was performed to assess the generalizability of threshold selection. We performed sensitivity analyses to assess the effects of segmentation algorithm and harmonization. Specifically, we repeated the above analysis using hippocampal volumes derived from the subfield module (instead of the aseg) and harmonized data (instead of unharmonized data). No cases were excluded in the analyses of unharmonized features. We excluded participants from the analyses of harmonized features if they were scanned with a protocol used in fewer than four participants (*N* = 9). In the IDEAS dataset, dual pathology is categorized separately from HS. As such, we performed an additional sensitivity analysis including patients with dual pathology in the HS group. All analyses were carried out using MATLAB 2025b (MathWorks, Natick, MA).

## Data availability

As described in the original publication,^10^ the data used in this manuscript are publicly available at: https://www.cnnp-lab.com/ideas-data. To facilitate validation, we developed an open-source software tool for calculation of hippocampal asymmetry index available at: https://phindagijimana.github.io/neuroinsight_landing_web/. Codes for analysis and figure generation can also be found at this address.

## Results

### Harmonization

**Figure 1:**
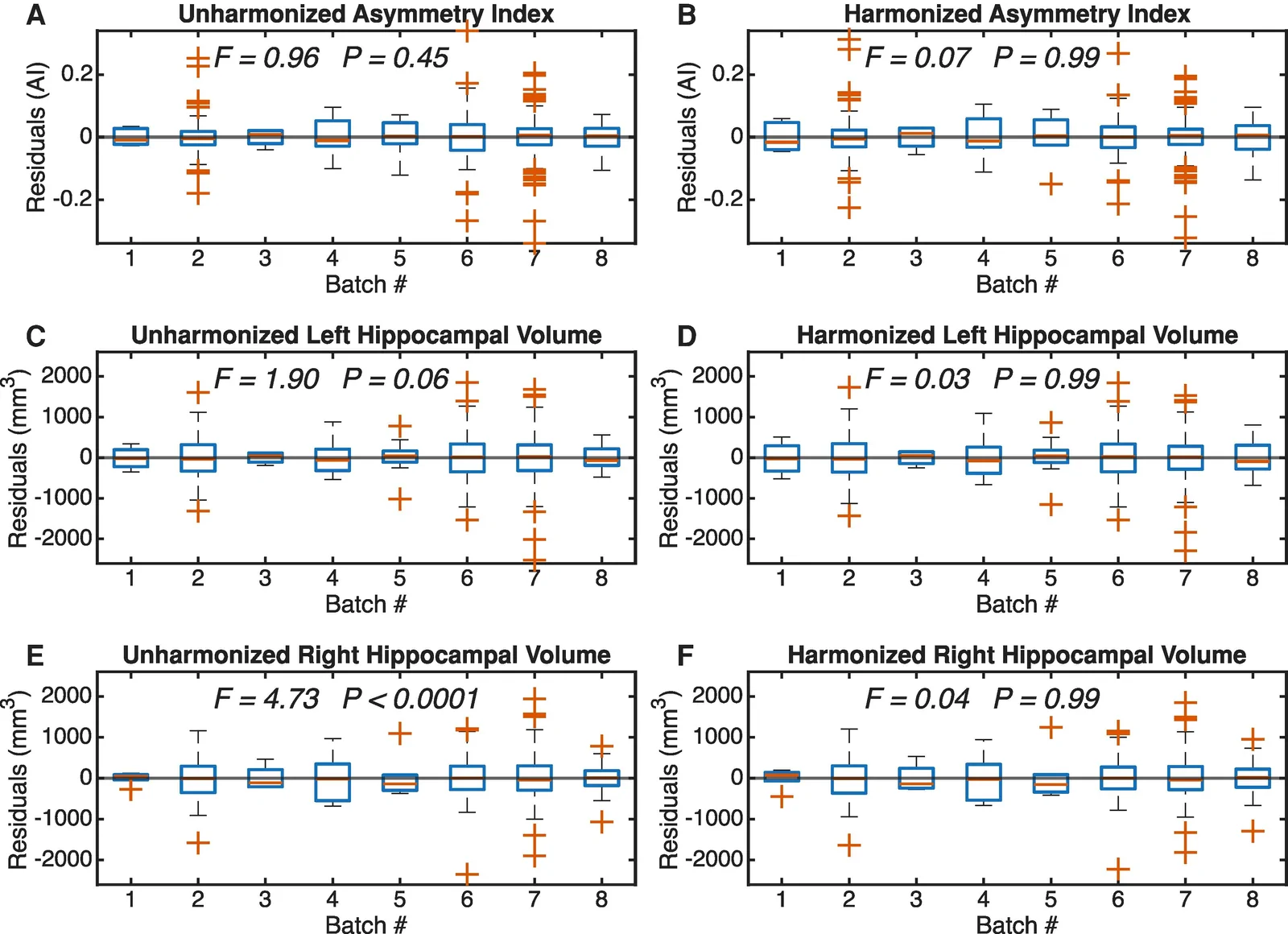
**Comparison of batch effects before and after harmonization.** Residuals for asymmetry and volume features from ANOVA models fit with biological covariates (age, sex, diagnosis) and batch. Data are grouped into eight batches (*x*-axis) representing unique combinations of scanner model and voxel size. Batches with a minimum of four participants are shown (*N* = 533). Residuals are plotted across the eight batches for asymmetry index (A, B) and volumes (C–F). Unharmonized data are shown in the left panel (A, C, E) and harmonized data on the right (B, D, F). For unharmonized data, there is strong evidence of batch effects in right hippocampal volume, but left hippocampal volume did not reach statistical significance (*P* = 0.06). Batch effects were not detectable in the unharmonized asymmetry index (*P* = 0.45). *P* < 0.05 is indicative of batch effects. Plus signs represent outlier values (i.e. values more than 1.5 times the interquartile range away from the top or bottom of the box).

There were eight unique combinations of scanner model and voxel size after removing batches with fewer than four participants (*n* = 533). Figure 1 shows the residuals for asymmetry and volume features from ANOVA models fit with biological covariates and batch. Batch effects were detectable in unharmonized volumetric data, but not in the unharmonized asymmetry index. There was evidence of unequal variance in both asymmetry index (*P* < 0.00001) and left hippocampal volume (*P* = 0.001) prior to harmonization. After harmonization, batch effects and unequal variance were no longer detectable in volumetric data (*P* > 0.05). These findings indicate the robustness of the asymmetry index, but not volumetric data, to site effects and differences in voxel volume.

### Diagnostic performance

The diagnostic performance of features derived from hippocampal volumetry in detection of HS pathology was high regardless of segmentation algorithm or whether harmonization was performed. The AUC of hippocampal asymmetry index for a pathologic diagnosis of left HS was 0.95 (95% confidence interval 0.92–0.97) and 0.96 (95% confidence interval 0.94–0.98) for right HS (Table 1). Normative asymmetry index and ipsilateral normative volumes did not improve diagnostic performance. Diagnostic performance was similar when using hippocampal volumes derived from the hippocampal subfield module (Supplementary Table 3). Diagnostic performance was not improved by using harmonization (Supplementary Tables 4 and 5). Diagnostic performance was similar when dual pathology was included in the HS group (Supplementary Table 6)

**Table 1:** Diagnostic performance for identification of pathologic diagnosis of HS using unharmonized hippocampal (aseg) volumes

	AUC (95% CI)	Sensitivity (95% CI)	Specificity (95% CI)
Left HS			
AI	0.95 (0.92–0.97)	0.95 (0.92–0.97)	0.9 (0.84–0.96)
Norm AI	0.95 (0.92–0.97)	0.96 (0.92–0.97)	0.89 (0.81–0.94)
Norm vol	0.9 (0.85–0.93)	0.93 (0.89–0.97)	0.75 (0.63–0.82)
Right HS			
AI	0.96 (0.94–0.98)	0.85 (0.68–0.91)	0.97 (0.94–0.98)
Norm AI	0.96 (0.94–0.98)	0.83 (0.67–0.88)	0.97 (0.95–0.99)
Norm vol	0.92 (0.87–0.95)	0.95 (0.91–0.97)	0.75 (0.59–0.85)

**Abbreviations:** AI, Asymmetry index; CI, confidence interval; HS, hippocampal sclerosis; Norm AI, Normative asymmetry index; Norm vol, Ipsilateral normative volume.

**Figure 2:**
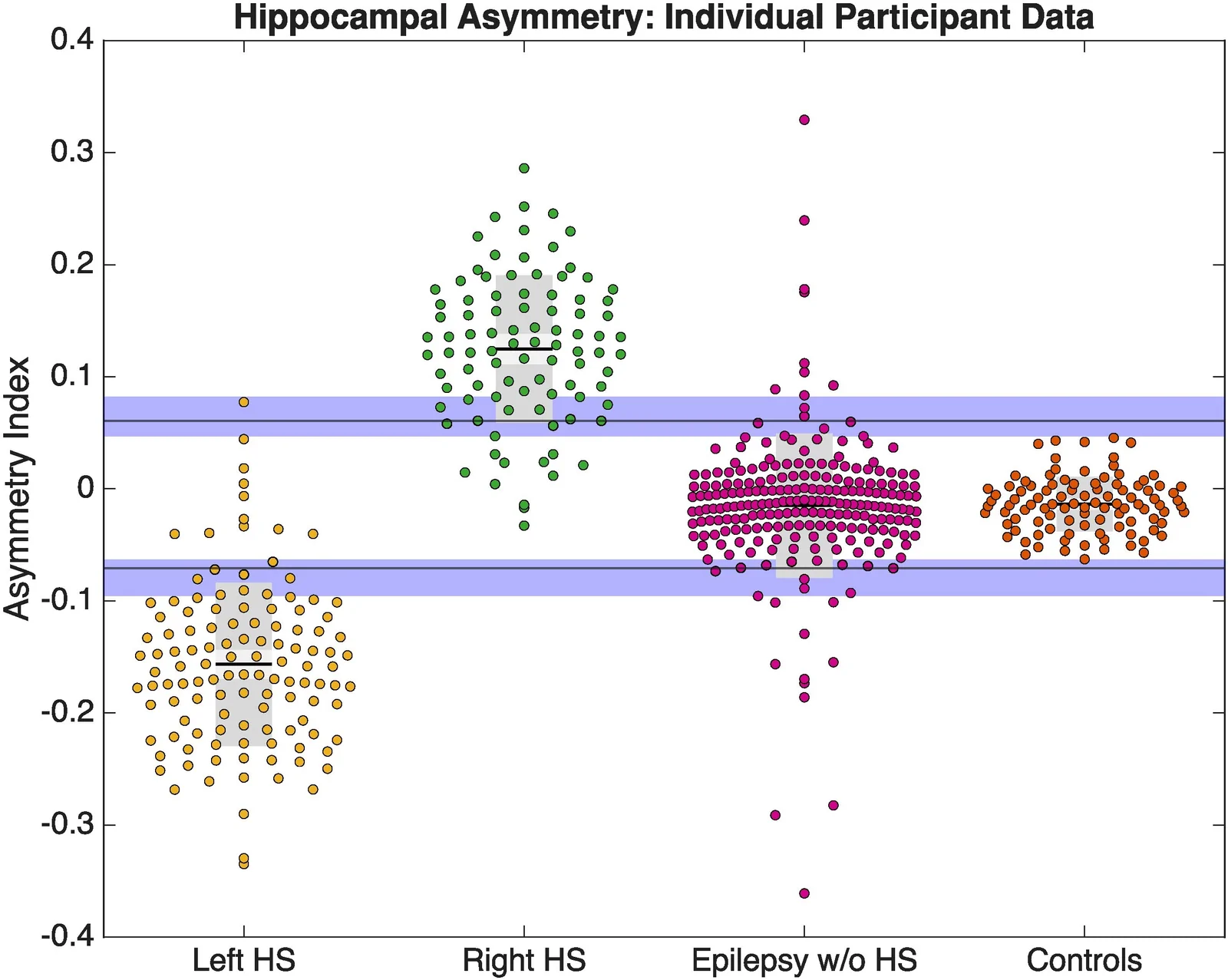
**Asymmetry Index across groups.** Unharmonized hippocampal asymmetry index is shown for epilepsy (*N* = 442) and controls (*N* = 100), with group means represented by solid horizontal black lines, standard deviation by dark shaded boxes, and standard error of the mean by light shaded boxes. The two horizontal lines represent the optimal operating point for detection of HS (upper right HS, lower left HS) with corresponding shaded areas representing confidence estimates determined using 5-fold cross-validation. Circles represent the study participant’s hippocampal volume asymmetry index. Abbreviations: HS, hippocampal sclerosis.

The diagnostic threshold for left HS was an asymmetry index of −0.071 and 0.061 for right HS, respectively. To assess the generalizability of these thresholds, we performed 5-fold cross-validation. The mean (range) threshold across folds was −0.078 (−0.096–0.063) and 0.064 (0.047–0.082) for left and right HS, respectively. These thresholds are displayed along individual participant data in Fig. 2. We did not detect any influence of age, sex, and intracranial volume on asymmetry index in healthy controls (Supplementary Figs 1–3).

## Discussion

In this study, we describe a simple method for identification of a pathologic diagnosis of HS using hippocampal asymmetry index derived from a patient’s clinically acquired pre-operative brain MRI. Unlike volumetric measures, batch effects were not detected in the hippocampal asymmetry index, indicating the robustness of the asymmetry index to differences in scanner models and voxel size. The hippocampal asymmetry index showed high diagnostic performance with sensitivities of 85–96% without harmonization compared to sensitivities of 83–96% with harmonization. Calculation of asymmetry index using normative data did not increase diagnostic performance. Similarly, normative ipsilateral hippocampal volumes did not improve upon diagnostic performance relative to asymmetry index. The approach described here represents a step towards identification of HS pathology using a patient’s pre-operative clinically acquired 3D T1-weighted image without the need for large normative datasets, which are typically not available outside the research setting. To facilitate validation, we provide an open-source software tool for calculation of hippocampal asymmetry index. This tool takes a 3D T1-weighted image as input, performs FreeSurfer reconstruction, calculates the hippocampal asymmetry index and labels the scan as consistent with either left HS, right HS, or neither based on the thresholds established in this study using unharmonized data.

This study builds upon several recent studies using features derived from clinically acquired brain MRI to identify HS. Ripart *et al*.^8^ developed a machine learning classifier using pre-operative T1-weighted images with a sensitivity of 90% for automatic detection of a histopathologic diagnosis of HS. Another study by Belke *et al*.^7^ developed a machine learning model trained on patients with radiologist-identified HS with sensitivities ranging from 73–98% in the validation cohort. Notably, both studies utilized healthy control data for feature generation. The use of normative or reference cohorts allows for estimation of hippocampal pathology but creates barriers for clinical translation. In contrast, our study showed comparable sensitivities ranging from 85–96% using only the patient’s T1-weighted image as input, obviating the need for control/reference data, machine learning methods susceptible to overfitting, and harmonization of multisite data.

This study has several notable limitations. A major limitation is the inability to identify bilateral HS cases. This retrospective cohort necessarily lacks details on bilateral HS because it only includes patients who underwent resective surgery. It is likely that many patients with bilateral HS were excluded from this cohort because they were deemed ineligible for surgery. There may also be patients with unrecognized bilateral HS that were missed when hippocampal pathology on the non-operative sign was MRI-occult. In bilateral HS, the asymmetry index may be normal if the degree of atrophy is more or less symmetric or in the case where there is significant volumetric asymmetry, the asymmetry index should point to the more atrophic hippocampus. Unfortunately, we cannot test this hypothesis in this cohort. Other potential failure modes include dual pathology and temporal lobe lesions that disrupt the architecture of the hippocampus. In the case of dual pathology, the hippocampal asymmetry index would not flag the second epileptogenic lesion. Extensive lesions may result in poor surface reconstruction and segmentation failure. Our software tool includes quality control images where the hippocampal segmentation labels are overlaid on the T1-weighted image. This allows the user to inspect the quality of the segmentation. In these cases, it would be particularly important to interpret the asymmetry index in the context of the patient’s larger presurgical evaluation. We envision that this tool, if validated, would be used as an adjunctive test in the presurgical evaluation of drug-resistant focal epilepsy where there may be evidence for bilateral mesial temporal lobe epilepsy or extrahippocampal pathology in other modalities such as bilateral independent temporal lobe or multifocal seizures, radiological signs of bilateral HS or extrahippocampal lesions, etc. Another limitation is the lack of availability of radiologists or multidisciplinary assessment of whether patients' MRIs were lesional or nonlesional. The asymmetry index may be particularly useful in cases where HS was MRI occult, but we were not able to address this question in this cohort. Nonetheless, we feel that a histopathologic diagnosis of HS should represent the gold standard given that histopathologic HS can be MRI occult (i.e. normal appearance of the hippocampus on MRI). Finally, the lack of an independent validation cohort represents a limitation. This is mitigated by the lack of model fitting and the large sample size with good representation of HS cases identified with gold standard histopathology. However, given that sensitivity and specificity are influenced by threshold selection, we performed 5-fold cross-validation to estimate a range of thresholds that may be seen with a true test set. Given that data in this cohort were acquired on a relatively constrained acquisition system (i.e., a single MRI vender), we could not assess robustness across other vendors, 1.5T systems, and different reconstruction pipelines.

In this study, we show that the hippocampal asymmetry index derived from clinically acquired pre-operative T1-weighted MRI can identify HS with high diagnostic performance. Moreover, this can be achieved without the need for a control/reference cohort, obviating the need for multisite data harmonization. This study, as well as other recent studies^7,8^ demonstrate that hippocampal asymmetry features derived from pre-operative MRI, if validated, have the potential for clinical translation, utilized alongside a typical clinical presurgical evaluation for drug-resistant focal epilepsy. To facilitate validation, we provide an open-source software tool that takes a 3D T1-weighted image as input, performs FreeSurfer reconstruction, and calculates the hippocampal asymmetry index.

## Supplementary Material

fcag320_Supplementary_Data
