# Supplementary material for: MRI derived hippocampal asymmetry identifies hippocampal sclerosis in epilepsy surgical specimens
Source: Brain Commun. 2026 Aug 21;8(4):fcag320. doi: 10.1093/braincomms/fcag320 (PMC13527870; doi:10.1093/braincomms/fcag320)
Supplement: Supplementary file 1 [file fcag320_supplementary_data.pdf]

Supplemental Table 1: Patient Demographics

		HS	Non-HS	P-value
Sex	Female	132 (61.4%)	106 (46.7%)	0.003
Age at seizure onset	Less than 1	10 (4.7%)	3 (1.3%)	<0.001
	1 to 2	20 (9.3%)	6 (2.6%)	
	3 to 4	16 (7.4%)	9 (4.0%)	
	5 to 7	30 (14.0%)	33 (14.5%)	
	8 to 10	32 (14.9%)	12 (5.3%)	
	11 to 14	31 (14.4%)	37 (16.3%)	
	15 to 19	32 (14.9%)	40 (17.6%)	
	20 to 24	17 (7.9%)	37 (16.3%)	
	25 to 29	10 (4.7%)	16 (7.0%)	
	30 to 34	6 (2.8%)	19 (8.4%)	
	35 to 39	8 (3.7%)	5 (2.2%)	
	Over 40	3 (1.4%)	10 (4.4%)	
FUS		208 (96.7%)	201 (88.5%)	0.002
FUS frequency		6.0 (3.0, 12.0)	9.0 (2.5, 28.0)	0.061
FBTCS		175 (81.4%)	168 (74.0%)	0.081
FBTCS frequency		0.1 (0.0, 1.0)	0.1 (0.0, 1.0)	0.366
Status epilepticus		33 (15.3%)	20 (8.8%)	0.049
Operative side	Left	122 (56.7%)	112 (49.3%)	0.143
	Right	93 (43.3%)	115 (50.7%)	
Operation type	F Lesx	0 (0.0%)	12 (5.3%)	<0.001
	F Lx	0 (0.0%)	46 (20.3%)	
	Hx	0 (0.0%)	2 (0.9%)	
	O Lesx	0 (0.0%)	2 (0.9%)	
	O Lx	0 (0.0%)	5 (2.2%)	
	O P Lx	0 (0.0%)	2 (0.9%)	
	P Lesx	0 (0.0%)	11 (4.8%)	
	P Lx	0 (0.0%)	6 (2.6%)	
	T Lesx	0 (0.0%)	37 (16.3%)	
	T Lx	215 (100.0%)	101 (44.5%)	
	T O Lesx	0 (0.0%)	1 (0.4%)	
	T P Lx	0 (0.0%)	2 (0.9%)	
Non-HS pathology	CAV		33 (14.5%)	
	DNT		52 (22.9%)	
	DUAL		21 (9.3%)	
	FCD		39 (17.2%)	

	GL		*5 (2.2%)*	
	OTHER		*75 (33.0%)*	
	TREBLE		*2 (0.9%)*	
HS pathology	*ILAE type 1*	*46 (21.4%)*		
	ILAE type 2	*8 (3.7%)*		
	ILAE type 3	*11 (5.1%)*		
	FCDIIIa	*11 (5.1%)*		
	Other	*12 (5.6%)*		
	NA	*133 (61.9%)*		
Number of ASMs		*3.0 (2.0, 3.0)*	*2.0 (2.0, 3.0)*	*0.074*
Age at scan	*15 to 20*	*11 (5.1%)*	*17 (7.5%)*	*0.048*
	20 to 24	*21 (9.8%)*	*37 (16.3%)*	
	25 to 29	*29 (13.5%)*	*44 (19.4%)*	
	30 to 34	*31 (14.4%)*	*38 (16.7%)*	
	35 to 39	*32 (14.9%)*	*30 (13.2%)*	
	40 to 44	*35 (16.3%)*	*22 (9.7%)*	
	45 to 49	*26 (12.1%)*	*14 (6.2%)*	
	50 to 54	*17 (7.9%)*	*14 (6.2%)*	
	55 to 59	*10 (4.7%)*	*6 (2.6%)*	
	60 to 64	*2 (0.9%)*	*2 (0.9%)*	
	65 to 70	*1 (0.5%)*	*3 (1.3%)*	
Age at surgery	*15 to 20*	*6 (2.8%)*	*7 (3.1%)*	*0.048*
	20 to 24	*19 (8.8%)*	*23 (10.1%)*	
	25 to 29	*24 (11.2%)*	*47 (20.7%)*	
	30 to 34	*33 (15.3%)*	*41 (18.1%)*	
	35 to 39	*30 (14.0%)*	*34 (15.0%)*	
	40 to 44	*31 (14.4%)*	*27 (11.9%)*	
	45 to 49	*33 (15.3%)*	*18 (7.9%)*	
	50 to 54	*22 (10.2%)*	*12 (5.3%)*	
	55 to 59	*11 (5.1%)*	*8 (3.5%)*	
	60 to 64	*5 (2.3%)*	*7 (3.1%)*	
	65 to 70	*1 (0.5%)*	*3 (1.3%)*	
ILAE year 1	*1*	*129 (61.1%)*	*116 (53.7%)*	*0.234*
	2	*24 (11.4%)*	*23 (10.6%)*	
	3	*27 (12.8%)*	*26 (12.0%)*	
	4	*24 (11.4%)*	*40 (18.5%)*	
	5	*7 (3.3%)*	*11 (5.1%)*	
ILAE year 2	*1*	*121 (59.6%)*	*117 (58.5%)*	*0.233*
	2	*17 (8.4%)*	*15 (7.5%)*	

	3	*25 (12.3%)*	*14 (7.0%)*	
	4	*31 (15.3%)*	*44 (22.0%)*	
	5	*9 (4.4%)*	*10 (5.0%)*	
ILAE year 3	*1*	*90 (50.6%)*	*97 (54.5%)*	*0.078*
	2	*16 (9.0%)*	*16 (9.0%)*	
	3	*29 (16.3%)*	*12 (6.7%)*	
	4	*35 (19.7%)*	*44 (24.7%)*	
	5	*8 (4.5%)*	*9 (5.1%)*	
ILAE year 4	*1*	*86 (54.8%)*	*82 (53.2%)*	*0.575*
	2	*10 (6.4%)*	*13 (8.4%)*	
	3	*20 (12.7%)*	*12 (7.8%)*	
	4	*32 (20.4%)*	*34 (22.1%)*	
	5	*9 (5.7%)*	*12 (7.8%)*	
	6	*0 (0.0%)*	*1 (0.6%)*	
ILAE year 5	*1*	*84 (59.2%)*	*69 (55.2%)*	*0.885*
	2	*10 (7.0%)*	*7 (5.6%)*	
	3	*13 (9.2%)*	*11 (8.8%)*	
	4	*26 (18.3%)*	*28 (22.4%)*	
	5	*9 (6.3%)*	*9 (7.2%)*	
	6	*0 (0.0%)*	*1 (0.8%)*	

Abbreviations: CAV, Cavernoma; DNT, Dysembryoplastic neuroepithelial tumor; Dual, Two pathologies; F, Frontal; FBTCS, History and monthly frequency of focal to bilateral tonic clonic seizures in 12 months pre-surgery; FCD, Focal Cortical Dysplasia; FUS, History and monthly frequency of focal unaware seizures in 12 months pre-surgery; GL, Glioma; HS, hippocampal sclerosis; Lx, Lobe resection; Lesx, Lesion resection; Hx, Hemispherectomy; O, Occipital; P, Parietal; T, Temporal; Treble, Three pathologies.

Supplemental Table 2: Scanner Information

Manufacturer Model	*Group count*	*Voxel volume, mean (SD)*	*Voxel volume range*
DISCOVERY MR750	*194*	*1.0062 (0.2176)*	*0.18-1.34*
SIGNA EXCITE	*92*	*0.9776 (0.0513)*	*0.97-1.32*
SIGNA HDx	*256*	*1.0066 (0.142)*	*0.97-2.64*

We assessed for differences in voxel volumes across the three different scanner models by fitting an ANOVA model with the manufacturer model as factor variable. We did not find systematic differences in voxel volume across scanner models (F = 1.1791, P = 0.30834).

Supplemental Table 3: Diagnostic performance for identification of pathologic diagnosis of HS using unharmonized volumes from the hippocampal subfield module

	AUC (95% CI)	*Sensitivity (95% CI)*	*Specificity (95% CI)*
Left HS			
AI	*0.95 (0.93-0.97)*	*0.96 (0.92-0.97)*	*0.91 (0.84-0.95)*
Norm AI	*0.95 (0.92-0.97)*	*0.96 (0.94-0.98)*	*0.91 (0.84-0.95)*
Norm vol	*0.92 (0.87-0.94)*	*0.91 (0.86-0.94)*	*0.82 (0.71-0.90)*
Right HS			
AI	*0.96 (0.94-0.98)*	*0.9 (0.76-0.97)*	*0.95 (0.92-0.97)*
Norm AI	*0.96 (0.93-0.98)*	*0.89 (0.76-0.97)*	*0.95 (0.91-0.97)*
Norm vol	*0.93 (0.88-0.95)*	*0.96 (0.93-0.99)*	*0.77 (0.60-0.86)*

Abbreviations: AI, Asymmetry index; CI, confidence interval; HS, hippocampal sclerosis; Norm AI, Normative asymmetry index; Norm vol, Ipsilateral normative volume.

Supplemental Table 4: Diagnostic performance for identification of pathologic diagnosis of HS using harmonized hippocampal (aseg) volumes

	AUC (95% CI)	*Sensitivity (95% CI)*	*Specificity (95% CI)*
Left HS			
AI	*0.95 (0.92-0.97)*	*0.96 (0.93-0.98)*	*0.87 (0.79-0.92)*
Norm AI	*0.95 (0.92-0.97)*	*0.96 (0.93-0.98)*	*0.88 (0.81-0.93)*
Norm vol	*0.9 (0.85-0.93)*	*0.92 (0.85-0.95)*	*0.78 (0.67-0.87)*
Right HS			
AI	*0.97 (0.94-0.98)*	*0.83 (0.71-0.88)*	*0.97 (0.96-0.99)*
Norm AI	*0.96 (0.94-0.98)*	*0.83 (0.7-0.89)*	*0.97 (0.95-0.99)*
Norm vol	*0.92 (0.88-0.95)*	*0.96 (0.92-0.97)*	*0.78 (0.68-0.88)*

Abbreviations: AI, Asymmetry index; CI, confidence interval; HS, hippocampal sclerosis; Norm AI, Normative asymmetry index; Norm vol, Ipsilateral normative volume.

Supplemental Table 5: Diagnostic performance for identification of pathologic diagnosis of HS using harmonized volumes from the hippocampal subfield module

	AUC (95% CI)	*Sensitivity (95% CI)*	*Specificity (95% CI)*
Left HS			
AI	*0.96 (0.93-0.98)*	*0.96 (0.93-0.98)*	*0.85 (0.76-0.91)*
Norm AI	*0.96 (0.93-0.98)*	*0.97 (0.95-0.99)*	*0.83 (0.74-0.88)*
Norm vol	*0.92 (0.88-0.94)*	*0.92 (0.88-0.96)*	*0.8 (0.66-0.88)*
Right HS			
AI	*0.96 (0.94-0.98)*	*0.84 (0.7-0.91)*	*0.96 (0.93-0.98)*
Norm AI	*0.96 (0.94-0.98)*	*0.85 (0.73-0.93)*	*0.96 (0.91-0.98)*
Norm vol	*0.93 (0.89-0.96)*	*0.96 (0.91-0.98)*	*0.76 (0.64-0.89)*

Abbreviations: AI, Asymmetry index; CI, confidence interval; HS, hippocampal sclerosis; Norm AI, Normative asymmetry index; Norm vol, Ipsilateral normative volume.

Supplemental Table 6: Diagnostic performance for identification of pathologic diagnosis of HS using unharmonized hippocampal (aseg) volumes. Dual pathology is included in the HS group.

	AUC (95% CI)	Sensitivity (95% CI)	Specificity (95% CI)
Left HS			
AI	*0.93 (0.90-0.96)*	*0.97 (0.93-0.98)*	*0.85 (0.78-0.91)*
Norm AI	*0.93 (0.89-0.96)*	*0.97 (0.94-0.99)*	*0.83 (0.75-0.89)*
Norm vol	*0.88 (0.83-0.91)*	*0.93 (0.87-0.96)*	*0.75 (0.65-0.84)*
Right HS			
AI	*0.95 (0.91-0.97)*	*0.81 (0.64-0.87)*	*0.97 (0.94-0.99)*
Norm AI	*0.94 (0.91-0.97)*	*0.79 (0.64-0.85)*	*0.97 (0.94-0.99)*
Norm vol	*0.88 (0.82-0.92)*	*0.95 (0.91-0.97)*	*0.71 (0.55-0.81)*

Abbreviations: AI, Asymmetry index; CI, confidence interval; HS, hippocampal sclerosis; Norm AI, Normative asymmetry index; Norm vol, Ipsilateral normative volume.

Supplemental Figure 1: Age Effects on Asymmetry Index

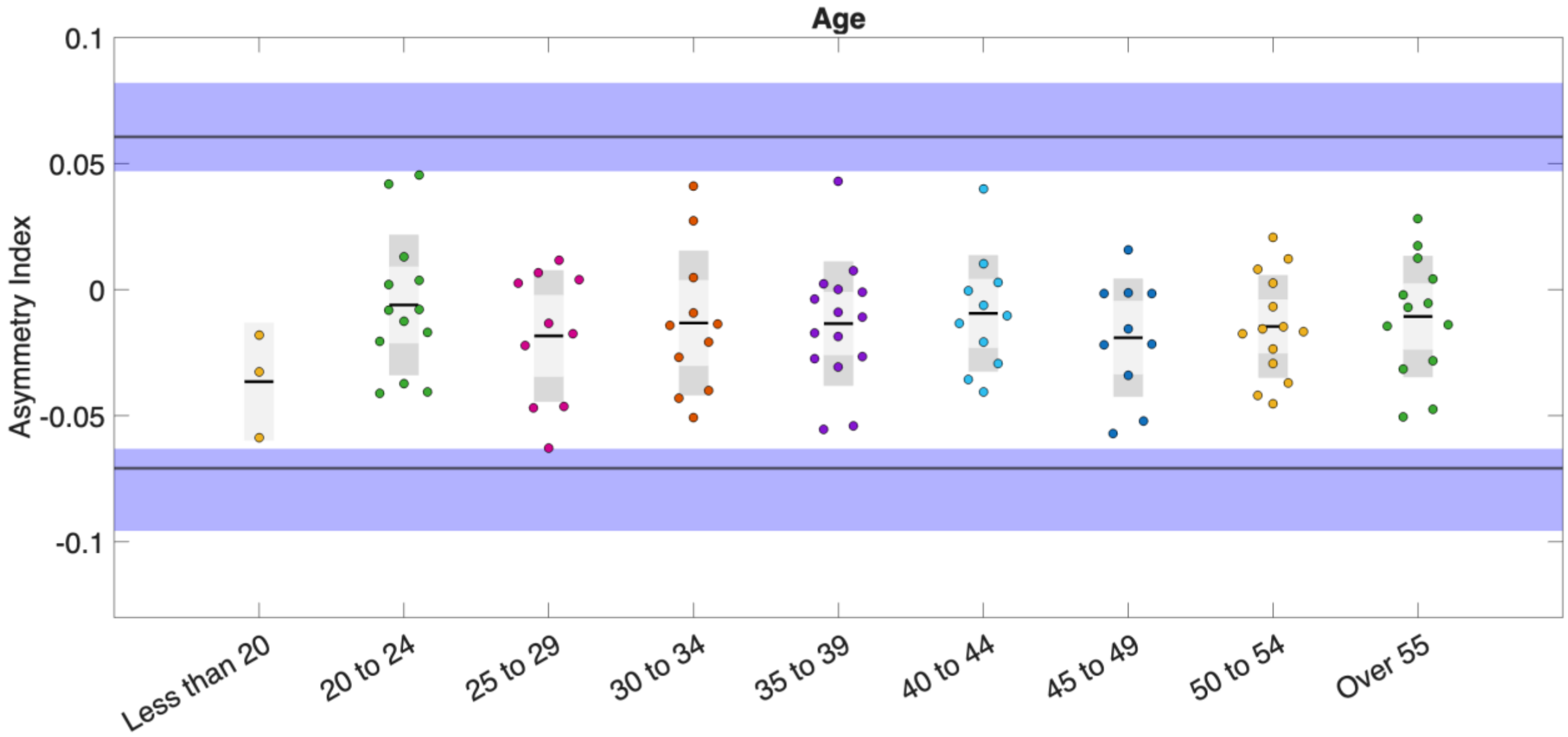

Caption: Unharmonized hippocampal asymmetry index derived from aseg volumes is shown for controls (N = 100) across age strata. Group means are represented by solid horizontal black lines, standard deviation by dark shaded boxes, and standard error of the mean by light shaded boxes. The gray horizontal lines represent the optimal operating point for detection of HS (upper right HS, lower left HS). Confidence estimates for optimal operating point are shown in blue and were determined using 5-fold cross validation. Circles represent control participant's hippocampal volume asymmetry index. Abbreviations: HS, hippocampal sclerosis.

Supplemental Figure 2: Sex Effects on Asymmetry Index

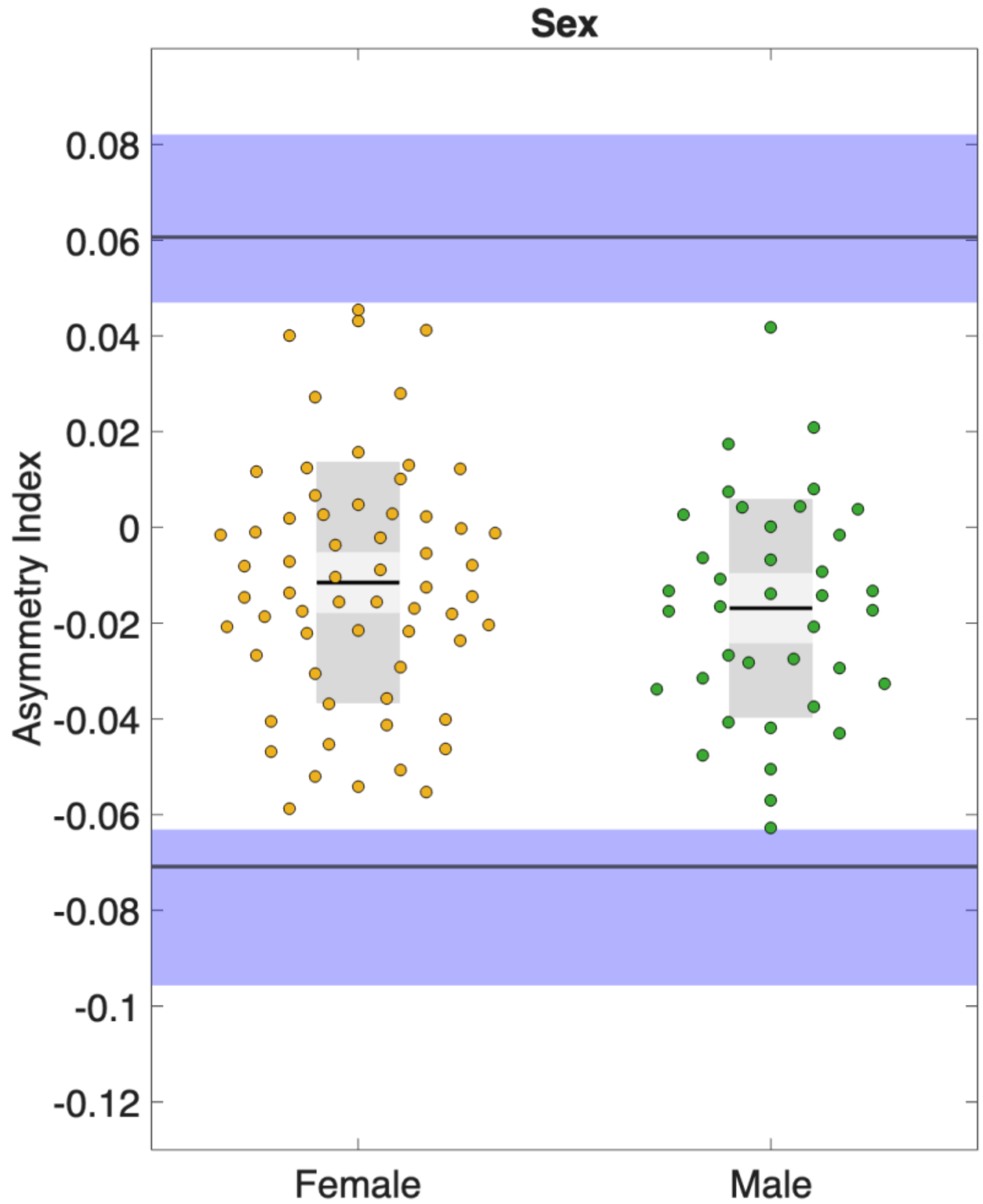

Caption: Unharmonized hippocampal asymmetry index derived from aseg volumes is shown for controls (N = 100) stratified by sex. Group means are represented by solid horizontal black lines, standard deviation by dark shaded boxes, and standard error of the mean by light shaded boxes. The gray horizontal lines represent the optimal operating point for detection of HS (upper right HS, lower left HS). Confidence estimates for optimal operating point are shown in blue and were determined using 5-fold cross validation. Circles represent control participant's hippocampal volume asymmetry index. Abbreviations: HS, hippocampal sclerosis.

Supplemental Figure 3: Asymmetry Index Across ICV

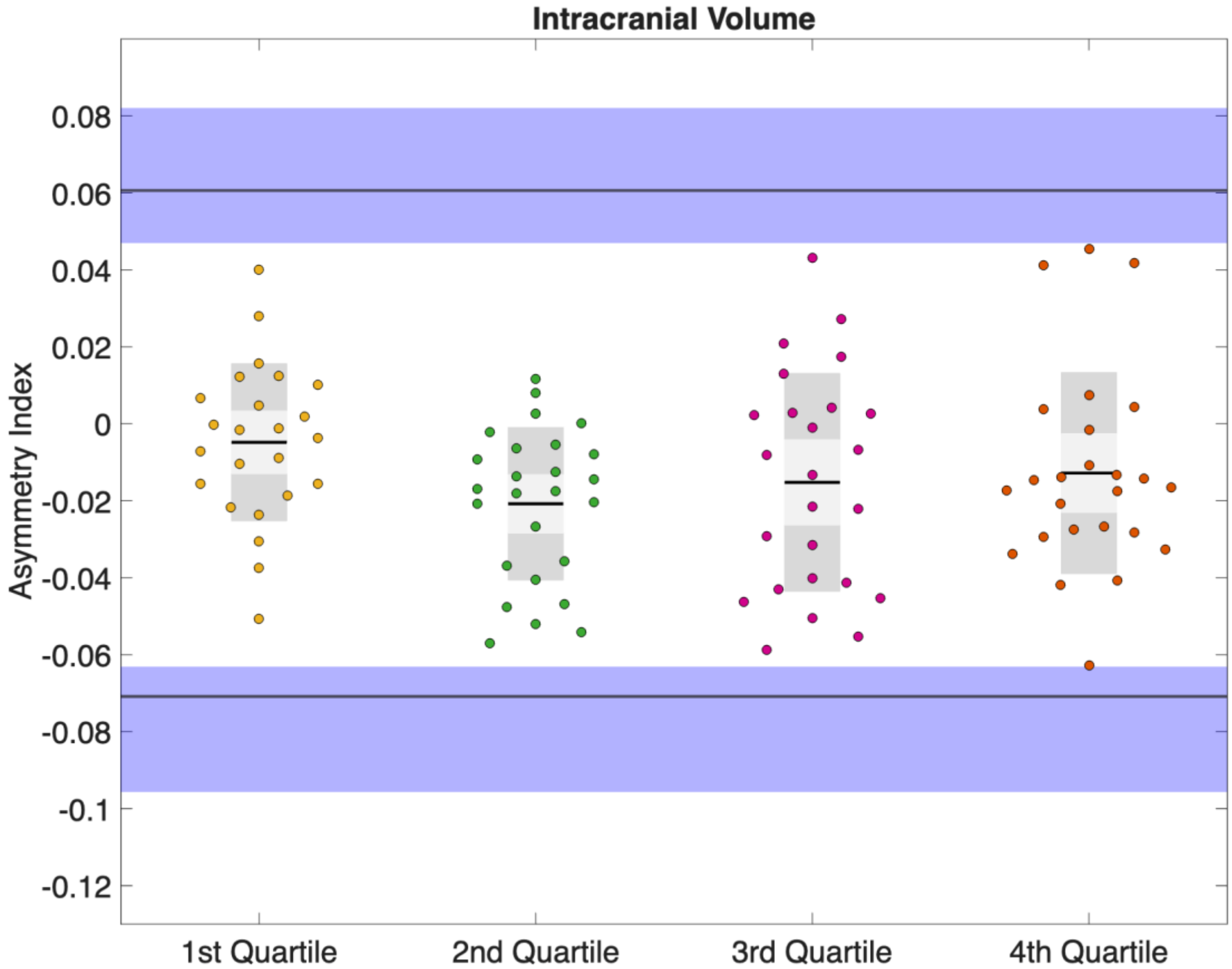

Caption: Unharmonized hippocampal asymmetry index derived from aseg volumes is shown for controls (N = 100) stratified by intracranial volume quartile. Group means are represented by solid horizontal black lines, standard deviation by dark shaded boxes, and standard error of the mean by light shaded boxes. The gray horizontal lines represent the optimal operating point for detection of HS (upper right HS, lower left HS). Confidence estimates for optimal operating point are shown in blue and were determined using 5-fold cross validation. Circles represent control participant's hippocampal volume asymmetry index. Abbreviations: HS, hippocampal sclerosis.

Supplementary Analysis:

Linear regression model predicting AI in controls:

$$AI \sim 1 + age + sex + estimated\ ICV$$

Variable	*Coefficient Estimate*	*P-Value*
Age	*0.00020665*	*0.84318*
Sex	*-0.003997*	*0.5283*
Estimated ICV	*-7.1423e-09*	*0.69645*
